# Authors' Reply: Interpreting Agreement in AI-Assisted American Society of Anesthesiologists (ASA) Classification

**DOI:** 10.2196/112521

**Published:** 2026-10-21

**Authors:** Bo-Han Lin, Cheng-Wei Lu, I-Ching Hou, Tzu-Yu Lin, Chin-E Liu, Chong-Sun Khoi

**Affiliations:** 1 College of Nursing and Smart Care Research Center, National Yang Ming Chiao Tung University, Taipei City, Taiwan; 2 Department of Anesthesiology, Far Eastern Memorial Hospital, No 21, Section 2, Nanya S Road, Banqiao District, New Taipei City, Taiwan, 886 2-8966-7000 ext 4371; 3 Department of Mechanical Engineering, College of Engineering, Yuan Ze University, Taoyuan City, Taiwan; 4 Graduate School of Biotechnology and Bioengineering, College of Medicine and Nursing, Yuan Ze University, Taoyuan City, Taiwan

**Keywords:** AI, American Society of Anesthesiologists, ASA, ASA physical status, preoperative assessment, agreement, Cohen κ

We thank the author for the thoughtful comments [1] on our study [2]. We provide supplemental analyses to clarify American Society of Anesthesiologists (ASA) classification agreement and the incremental contribution of Taiwan National Health Insurance (NHI) data.

First, the original analysis used the unweighted Cohen κ to evaluate the exact agreement between AI-generated and clinician-assigned ASA classifications in routine preanesthetic documentation. This statistic treats any category mismatch as a disagreement while accounting for agreement expected by chance. We acknowledge that the absence of weighting should have been explicitly stated in the Methods.

Recognizing the ordinal nature of ASA classification, we additionally calculated the linearly weighted Cohen κ, which was 0.893 with NHI data and 0.560 without NHI data.

We also examined patient-level cross-tabulations. With NHI data, among 57 clinician-assigned ASA III patients, AI classified 50 as ASA III and 7 as ASA II; among 6 ASA IV patients, AI classified 4 as ASA IV and 2 as ASA III. Without NHI data, among clinician-assigned ASA III patients, AI classified 44 as ASA III, 12 as ASA II, and 1 as ASA IV; among ASA IV patients, AI classified 2 as ASA IV, 3 as ASA III, and 1 as ASA II. Among these 63 patients, all 9 disagreements with NHI data involved adjacent categories. Without NHI data, 16 of 17 disagreements involved adjacent categories, and 1 ASA IV patient was classified as ASA II.

Second, we estimated uncertainty in the difference in unweighted κ using 10,000 patient-level paired bootstrap replicates. In each replicate, 600 patients were sampled with replacement, retaining each patient’s clinician-assigned ASA classification and both AI classifications together. Both unweighted κ estimates and their differences were recalculated in each replicate, accounting for the dependence between conditions.

The published unweighted κ estimates remain 0.883 (95% CI 0.841-0.946) with NHI data and 0.518 (95% CI 0.412-0.691) without NHI data. The observed difference was Δκ=0.365, with a supplemental 95% percentile bootstrap CI of 0.292-0.440, derived from the 2.5th and 97.5th percentiles of the bootstrap distribution of paired differences. This interval quantifies the precision of the estimated increase and excludes zero, supporting higher overall agreement when NHI data were included.

These supplemental results clarify both disagreement patterns and uncertainty in the NHI comparison. Nevertheless, adjacent-category discrepancies may still be clinically relevant. The predominance of ASA II patients and small ASA III to IV subgroups limit category-specific interpretation; neither unweighted nor weighted κ establishes equivalent performance across ASA categories. Comparisons with previous interrater studies also require caution because of differences in rater design, clinical setting, case mix, and κ weighting. We appreciate the opportunity to clarify these findings.

## Abbreviations

ASA: American Society of Anesthesiologists
NHI: National Health Insurance
